# Platelet Src family kinases: A tale of reversible phosphorylation

**DOI:** 10.1002/rth2.12495

**Published:** 2021-03-26

**Authors:** Yotis A. Senis, Zoltan Nagy, Jun Mori, Sophia Lane, Patrick Lane

**Affiliations:** ^1^ Unité Mixte de Recherche‐S 1255 Fédération de Médecine Translationnelle de Strasbourg Université de Strasbourg Institut National de la Santé et de la Recherche Médicale Etablissement Français du Sang Grand Est Strasbourg France; ^2^ Institute of Experimental Biomedicine University Hospital and Rudolf Virchow Center University of Würzburg Würzburg Germany; ^3^ Research and Development Align Technology Inc. Yokohama Japan; ^4^ Illustration and Design, ScEYEnce Studios Elkins Park PA USA

**Keywords:** kinase, phosphatatase, platelets, Src, tyrosine phosphorylation

## Abstract

Sarcoma (Src) family kinases (SFKs) have occupied a central place in platelet research for over 40 years. Discovered by virologists and oncologists as the proto *proto‐oncogene*, Src tyrosine kinase spurred a phenomenal burst of research on reversible tyrosine phosphorylation and signal transduction. For a time, platelets were adopted as the model of choice for studying the biological functions of Src, owing to their ease of isolation, high Src expression, and lack of a nucleus, only to be abandoned due to challenges of culturing and manipulating using common molecular biology‐based techniques. For platelet biologists, SFKs have remained an important area of investigation, initiating and amplifying signals from all major adhesion, activation, and inhibitory receptors, including the integrin αIIbβ3, the collagen receptor complex glycoprotein VI–Fc receptor γ‐chain, the G protein–coupled ADP receptor P2Y_12_ and the inhibitory receptors platelet endothelial cell adhesion molecule‐1 and G6b‐B. The vital roles of SFKs in platelets is highlighted by the severe phenotypes of *null* and *gain‐of‐function* mutations in SFKs in mice and humans, and effects of pharmacologic inhibitors on platelet activation, thrombosis, and hemostasis. The recent description of critical regulators of SFKs in platelets, namely, C‐terminal Src kinase (Csk), Csk homologous kinase (Chk), the receptor‐type protein‐tyrosine phosphatase receptor type J (PTPRJ) helps explain some of the bleeding side effects of tyrosine kinase inhibitors and are novel therapeutic targets for regulating the thrombotic and hemostatic capacity of platelets. Recent findings from Chk, Csk, and PTPRJ knockout mouse models highlighted that SFKs are able to autoinhibit by phosphorylating their C‐terminal tyrosine residues, providing fundamental insights into SFK autoregulation.

**Essentials**

- Sarcoma (Src) family kinases (SFKs) are essential for initiating and amplifying platelet activation.
- Reversible phosphorylation is a primary mode of regulation of SFK activity.
- The tyrosine kinases C‐terminal Src kinase (Csk) and Csk homologous kinase and phosphatase protein‐tyrosine phosphatase receptor type J are critical regulators of SFKs.
- Autophosphorylation provides an additional level of SFK regulation.

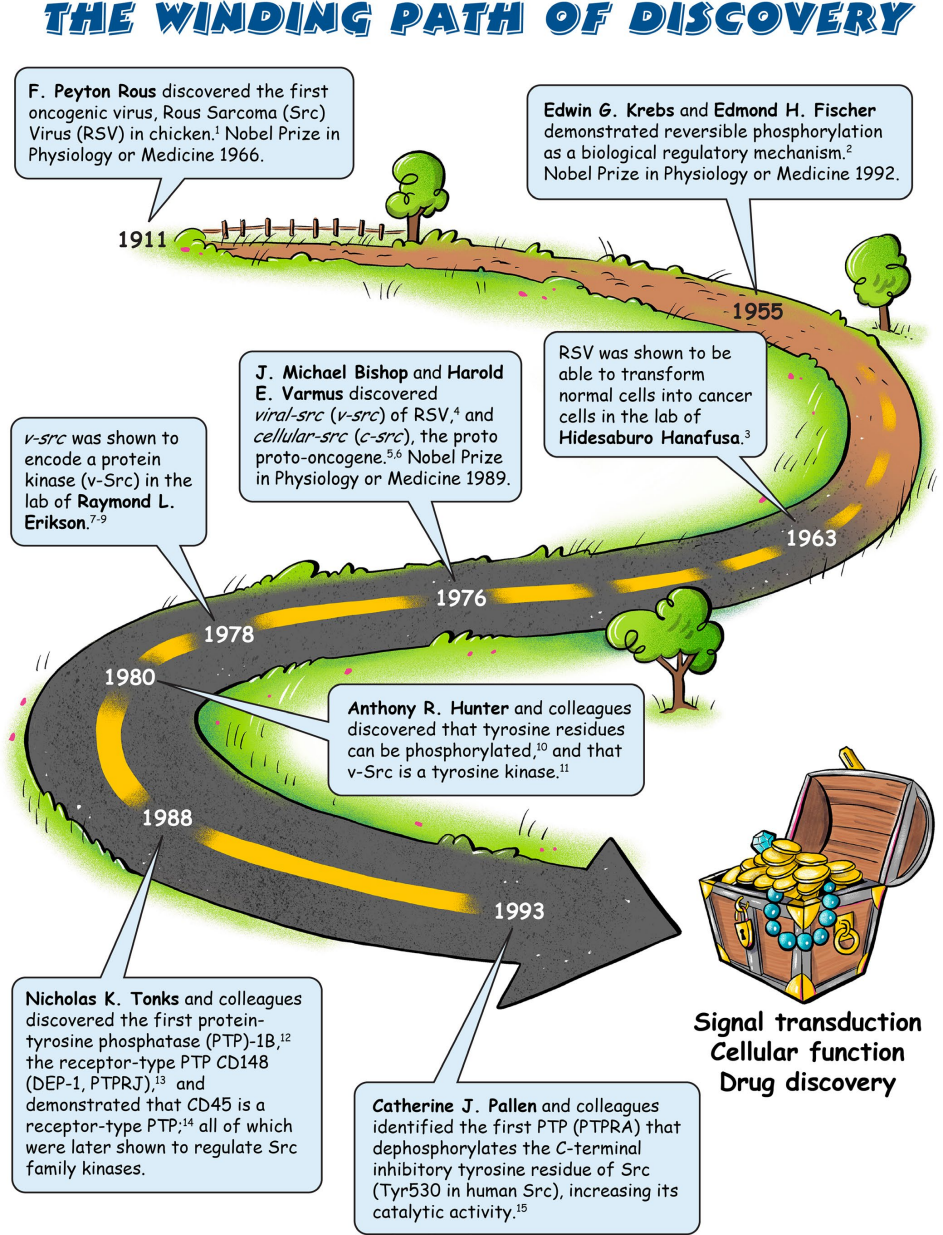

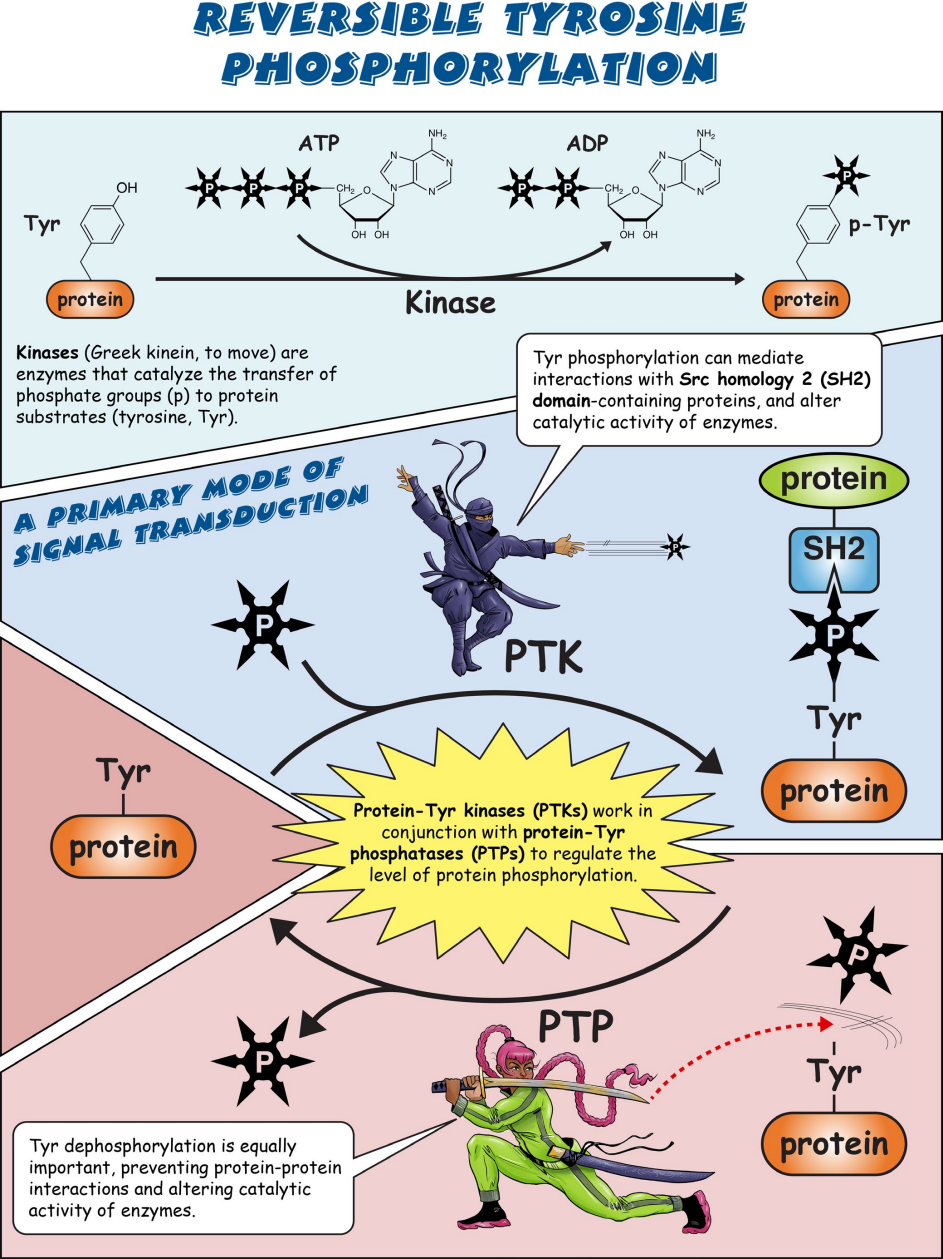

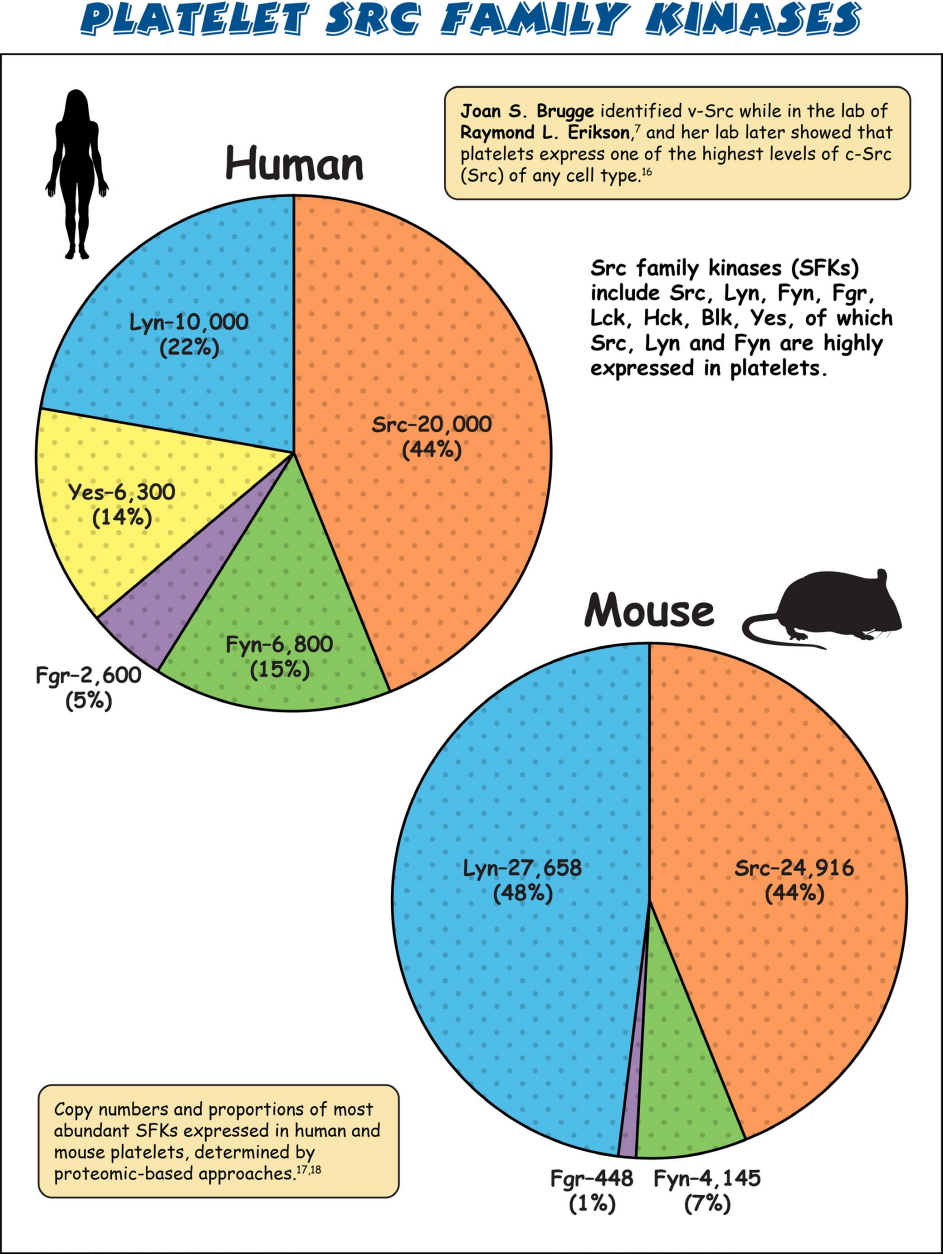

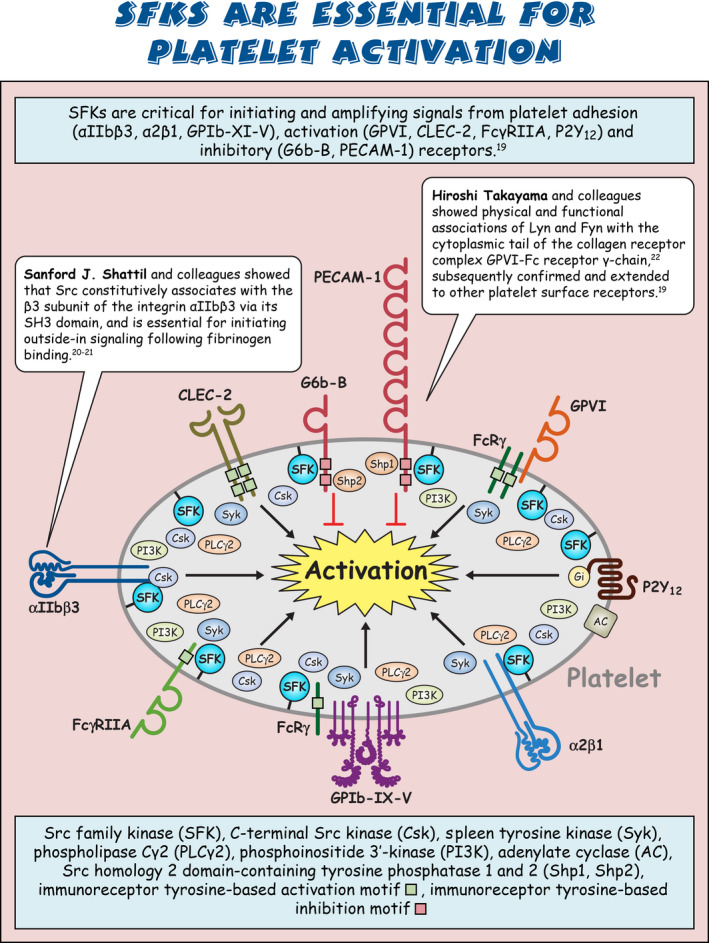

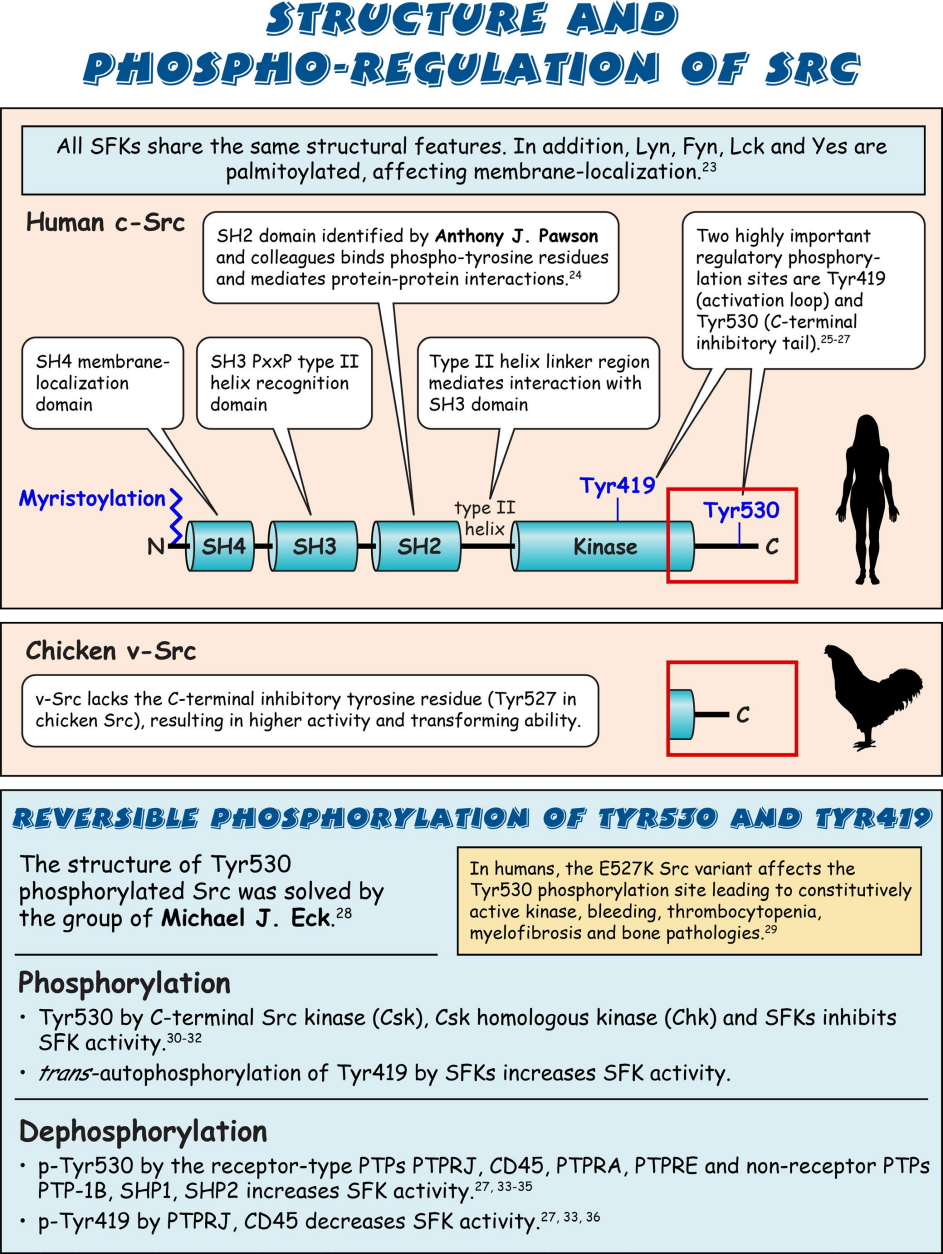

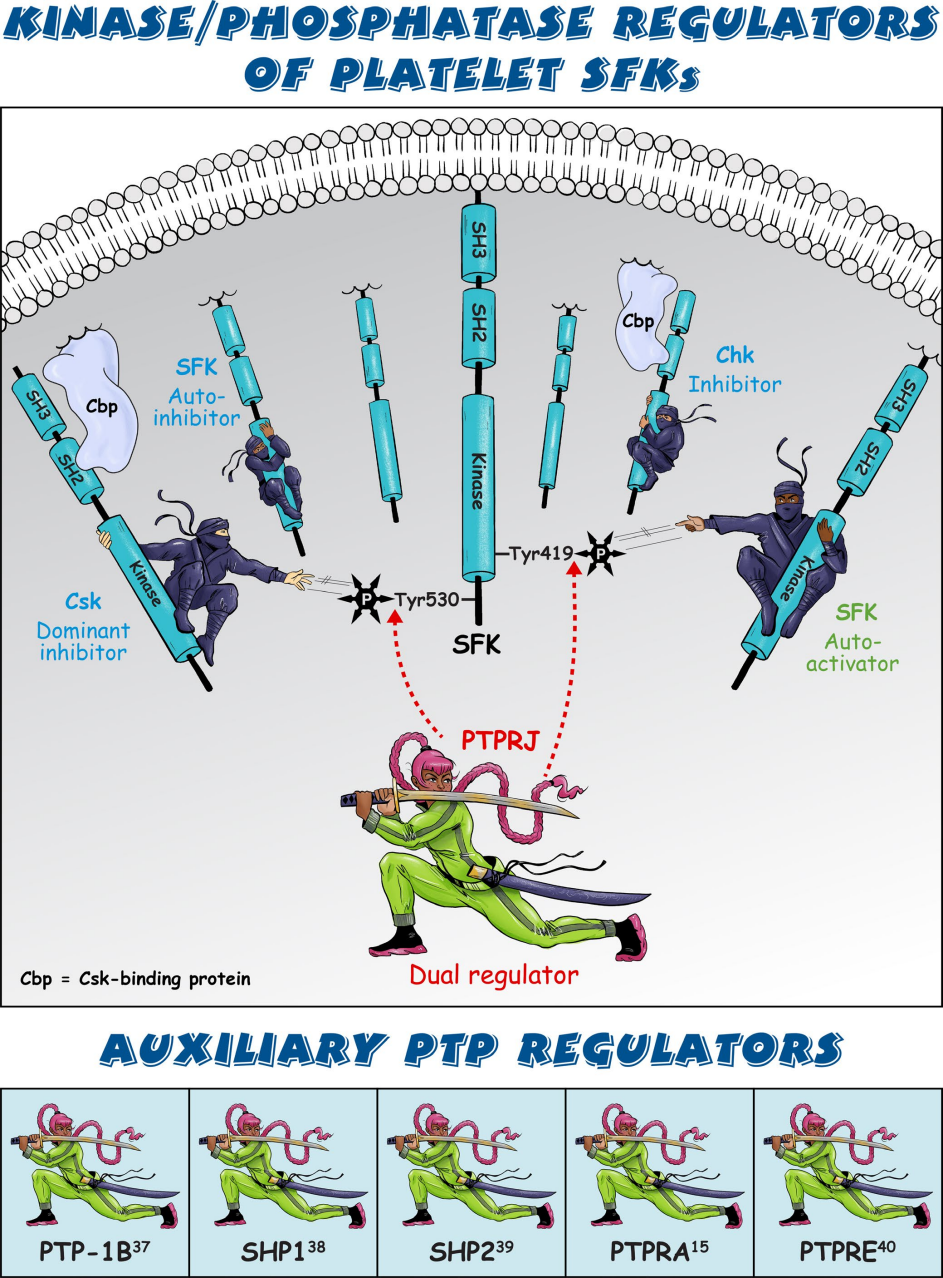

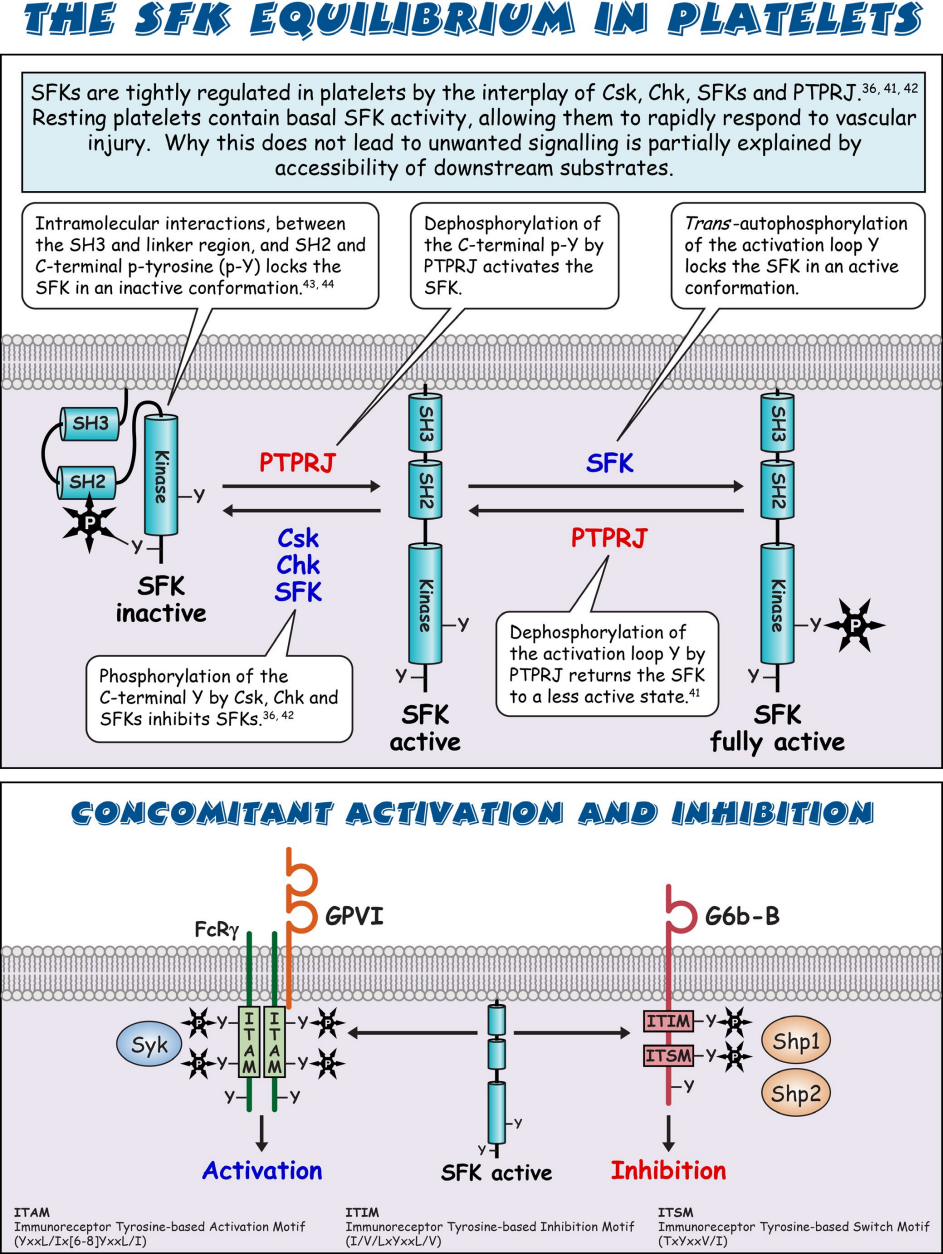

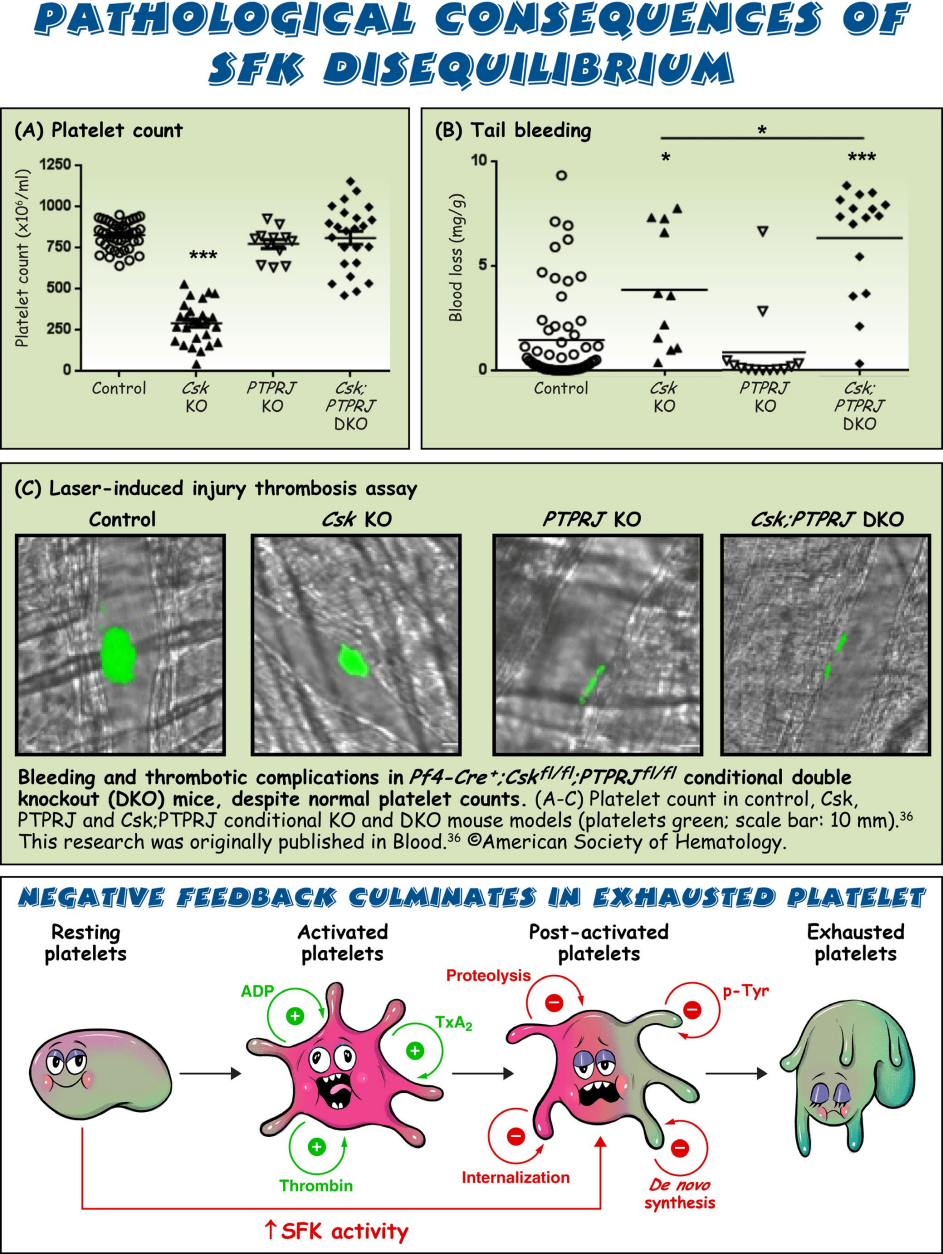

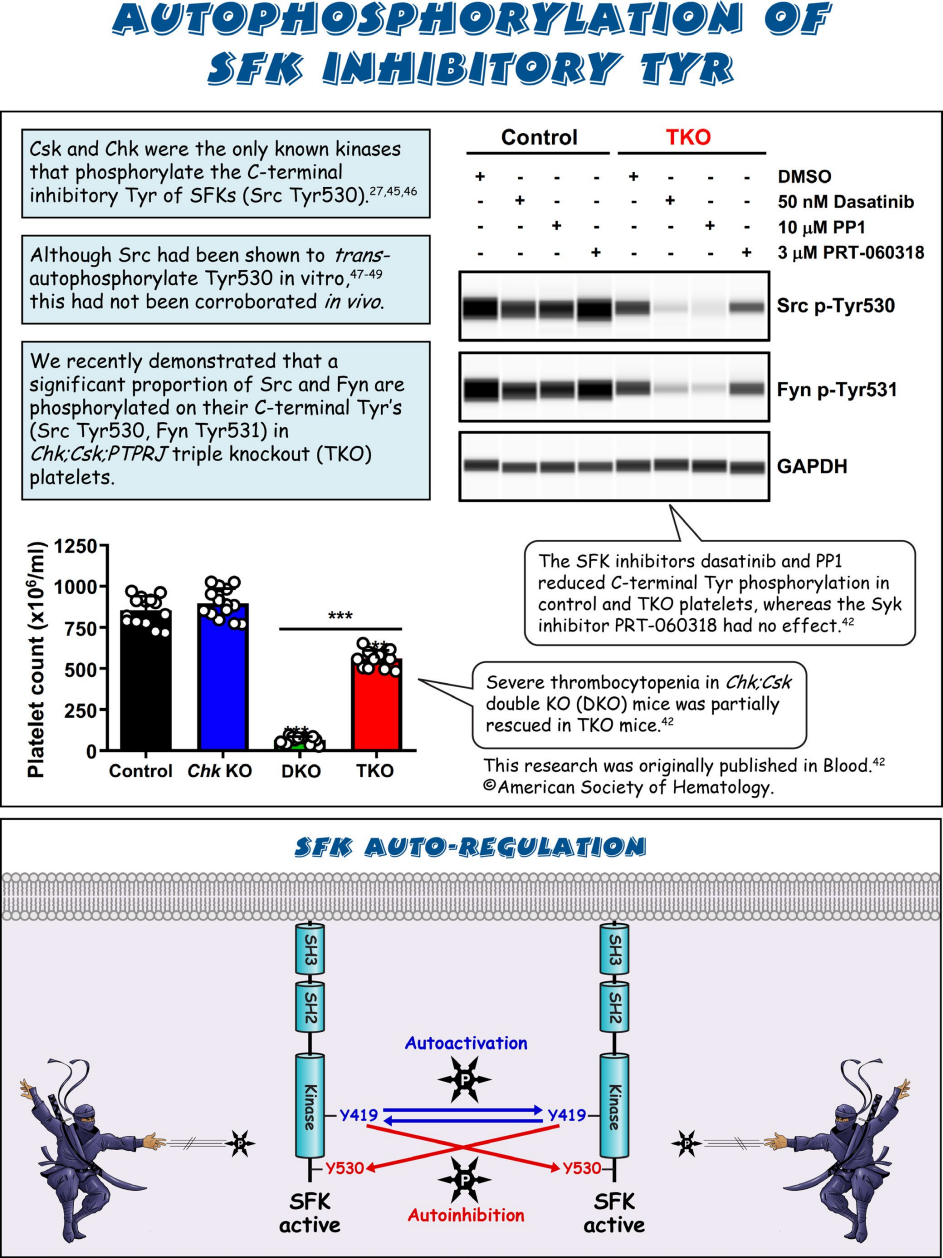

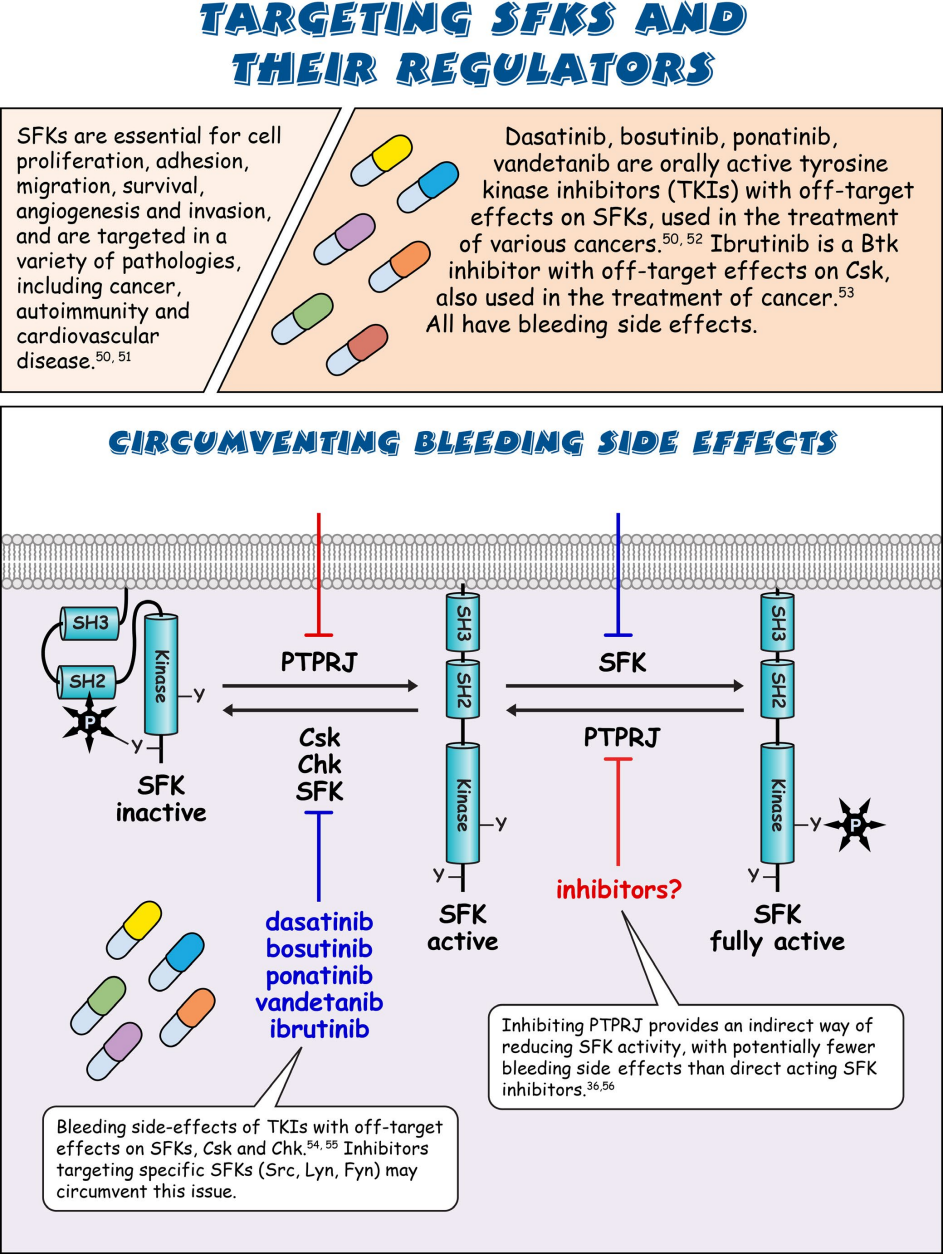

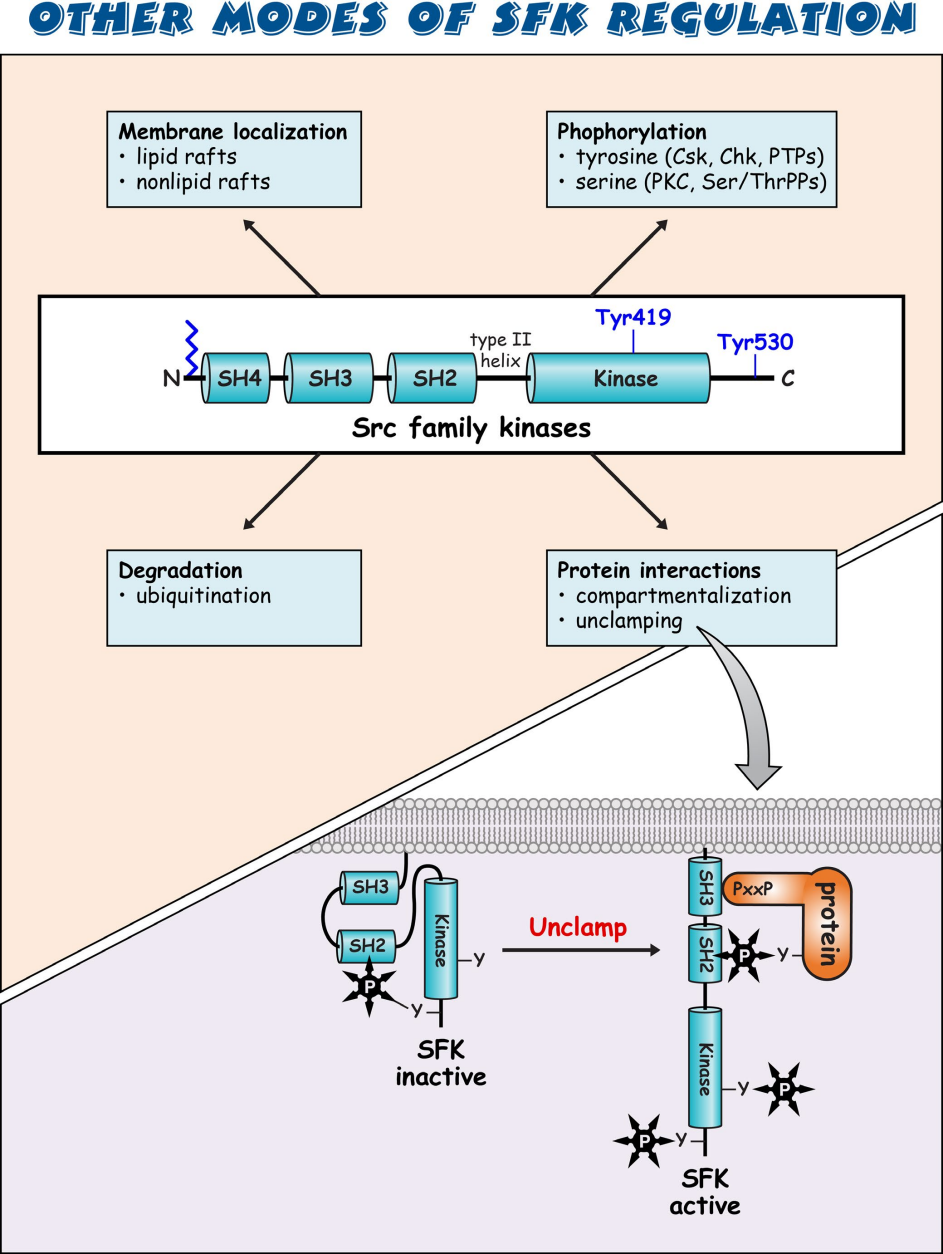

## AUTHOR CONTRIBUTIONS

YAS developed theme, prepared images, and wrote and revised the manuscript. ZN prepared images and revised the manuscript. JM developed theme and prepared images. SL developed theme, illustration, and design. PL developed theme, illustration, and design.

## RELATIONSHIP DISCLOSURE

The authors report no conflicts of interest to disclose.
